# Chorioretinal Alterations Induced by Preeclampsia

**DOI:** 10.1155/2021/8847001

**Published:** 2021-03-10

**Authors:** Xinyi He, Yimei Ji, Meiting Yu, Yuhua Tong

**Affiliations:** ^1^Second Clinical Medical College, Zhejiang Chinese Medical University, Hangzhou, Zhejiang 310053, China; ^2^Department of Ophthalmology, People's Hospital of Quzhou, Quzhou, Zhejiang 324000, China; ^3^Department of Gynecology and Obstetrics, People's Hospital of Quzhou, Quzhou, Zhejiang 324000, China

## Abstract

Hypertension during pregnancy, which is essentially a microvascular disease that destroys the end-organ microcirculation, should not be underestimated, as it could lead to organ failure in the kidneys, lungs, and brain. Preassessment of the microcirculatory state through systematic observation of the fundus has been proven to be noninvasive and feasible. Although hypertension in preeclampsia patients will resolve after childbirth, the sticking point is determining the best termination moment. Early diagnosis and treatment can prevent long-term ocular complications and cardiovascular risks for pregnant women in the future. In order to adjust the treatment strategy through more sensitive and precise fundus changes, we comprehensively summarized the common structural changes in the fundus in preeclampsia patients, including changes in the blood vessels, choroid, and retina, as well as the application of quantitative observation for chorioretinal alterations in recent years.

## 1. Introduction

Hypertensive disease in pregnancy, the most common disease worldwide, is responsible for significant morbidity and mortality for both mothers and children. The clinical syndrome is ascribed to maternal systemic microvascular disturbance, which affects all organs throughout the body.

One of the organs affected is the eyes. The disease can generate ocular alterations affecting the conjunctiva, choroid, and retina [1]. The choroid is one of the most vascularized tissues in the human body, whose distinctive role is to supply oxygen and nutrients to the outer retina. Consequently, chorioretinal changes can be affected by the microcirculation. Once the choroid blood supply is impaired, the retina will also suffer a corresponding disorder.

Complex interactions exist between the ocular impact on preeclampsia (PE) and how ocular dysfunction changes the process of pregnancy.

The aim of this study was to review the current literature concerned with fundus alterations in PE. The following paragraphs will systematically introduce PE, the anatomy and physiology of the fundus, how PE changes the fundus, vascular changes in the retina, hypertensive choroidopathy, and hypertensive retinopathy.

## 2. Methods

The related literature was searched from 2000 to 2020 in PubMed and the Web of Science database. The search strategy was based on a combination of terms: (1) “pre-eclampsia” or “preeclampsia” or “pregnancy toxemias;” (2) “diseases, retinal” or “retinal disease” or “retinopathy;” (3) “choroid” or “choriocapillaris” or “choroid disease.” The duplicate articles were removed.

### 2.1. PE

PE is a multisystemic disorder that is defined as new-onset hypertension and proteinuria during pregnancy after 20 weeks. However, according to the literature, patients with or without proteinuria seem to be untreatable if there is evidence of systemic involvement, such as thrombocytopenia, renal insufficiency, or pulmonary edema [2]. Moreover, compared with women without proteinuria, women with proteinuria are at greater risk of severe hypertension and of delivering premature babies or babies younger than their gestation period [3].

PE can deteriorate rapidly without any warning and is closely related to the prognosis of the mother and fetus. Sixteen percent of maternal deaths worldwide can be attributed to hypertension, resulting in more than 70000 maternal and over 500000 fetal and neonatal deaths each year [4]. Risk factors include maternal age, multiple pregnancies, childbirth, and the history of complicated systemic diseases.

The etiology and pathogenetic mechanisms of PE remain controversial. Numerous factors give rise to gestational hypertension, including heredity, hypoxia, trophoblast invasion, oxidative stress, and intravascular inflammation. However, it is generally accepted that the disease originates from reduced placental perfusion, which leads to abnormal vascular remodeling and maternal vascular endothelial dysfunction resulting in multiple organ failure in PE [1, 5–11].

The short-term complications of PE include cerebrovascular accidents, acute renal failure, and even maternal death. Previous literature indicates that long-term risks and mortality are increasing in women with PE [12, 13]. Further research is needed to advance the current knowledge to decrease the incidence of adverse perinatal outcomes, including fetal growth restriction and stillbirth.

### 2.2. Anatomy and Physiology of the Fundus

The retina has a dual blood delivery system: the central retinal artery (CRA) is the terminal vasculum, which mainly supplies the inner five layers of the retina; the other is the choroidal vascular system, which serves the outer five layers, and is composed of the two main posterior ciliary arteries (PCAs), namely, the medial and lateral PCA.

The choroid is mainly a vascular network that nourishes the eyeball and regulates blood volume. Traditionally, the choroid is viewed as vascularized tissue that comprises several vascular layers. Judging by the vascular thickness and density, from inside to outside, they are defined as the Haller layer, Sattler layer, and capillary layer, respectively. The Haller layer contains thick choroidal vessels, while the Sattler layer contains medium-sized blood vessels. The choroid provides oxygen and nutrients for the retinal pigment epithelium (RPE), outer retina, optic nerve, and avascular fovea. Other functions probably include light absorption, heat dissipation to regulate body temperature, and blood flow control through vasomotor and intraocular pressure (IOP).

### 2.3. How PE Changes the Fundus

The retina shows no physiological or visible changes in the fundus during normal pregnancy. However, almost all pregnant women have reactive changes in the retinal arterioles; one study showed that significant changes occurred only during PE or eclampsia seizures [14].

PE is routinely considered to be a vascular disorder. Hypertension in pregnancy exerts a striking effect on capillaries flowing through the organs. Studies have stated that 40–100% of PE pregnancies have retinal vascular abnormalities, which are regarded as indicators of termination of the pregnancy. Some potent endothelium-derived vasoconstrictors produced by the maternal placenta may induce severe spasms in the vessels of the brain, spleen, kidneys, and lungs. Due to the upregulation of the sympathetic nervous system in PE, the retinal blood vessels are not dominated by sympathetic nerves. Therefore, observations of the fundus have become an ideal choice for the direct and objective assessment of systemic microvascular changes during pregnancy.

Some studies have reported that choroidal ischemia and retinal circulation disorders are present in 30–100% of PE patients [15]. Choroidal perfusion is highly sensitive to abnormalities in systemic blood flow and perfusion. Hemodynamic changes during pregnancy may provoke pregnancy-related fluid retention in the choroidal layer, leading to increases in choroidal thickness. However, PE is often accompanied by systemic vasospasm. The main arterioles of the eye contract and spasm, including the PCAs and the CRA, result in a corresponding decrease in the choroidal blood supply. Moreover, systemic vascular resistance, including in the choroidal vessels, is increased in PE patients, which critically reduces choroidal vascular perfusion and presents a thinner choroid than that in healthy pregnancy patients.

The normal function of the retina depends on the normal morphological structure of the choroid. Once damage occurs in the RPE and blood-retinal barrier, protein and fluid leakage into the retinal neuroepithelial layer is inevitable, which ultimately causes retinal exudation, edema, and detachment. In addition, if the arterioles suffer hypoxia to a certain degree, arteriosclerosis is progressively promoted, which aggravates the interruption of blood flow, leading to the eventual occurrence of retinal hypoxic ischemic necrosis. The increased permeability in the hardened arterioles is the original cause of retinal bleeding, exudation, edema, and even retinal detachment in some severe cases. The chorioretinal abnormalities caused by PE mainly include segmental and generalized arterial stenosis, choroidal vascular network ischemia, retinal bleeding, central serous chorioretinopathy (CSC), and serous retinal detachment (SRD).

### 2.4. Vascular Changes in the Retina

Retinal vascular abnormalities caused by PE are similar to those in chronic hypertensive retinopathy. The most common retinal abnormalities are focal arteriole spasm and narrowing. Ophthalmological evaluations of PE patients have been mostly obtained through fundoscopy. Fundus photography mainly captures evident and visible retinal alterations, such as artery stenosis, for obstetric treatment and intervention. However, direct fundoscopy has proven to be unreliable due to its subjectivity, with a high rate of variation between observers (20–40%) and by the observers themselves (10–33%) [16, 17].

A genome-wide linkage analysis from the Beaver Dam Eye Study established a relationship between the diameter of retinal arterioles and multiple gene loci that are linked with the regulation of blood pressure, endothelial function, and angiogenesis [18], which suggests that retinal arteriole stenosis may be a surrogate marker of the individual genetic predisposition to hypertension [19, 20]. Ramos found that novel vascular interstitial cells (VICs) exist between endothelial and smooth muscle cells, allowing these cells to sense changes in vascular tension [21]. VICs have the ability to synthesize nitric oxide (NO), potentially due to its NADPH-diaphorase activity, thereby involving them in the regulation of blood pressure and local blood flow. Generally, VICs exert sphincter-like activities during hypertension to adjust retinal blood flow.

When the lesion becomes macroscopically visible, an irreversible loss of pathological structure and function has occurred. Therefore, highly sensitive technology is needed in the diagnosis and prognosis of chorioretinal disorders in pregnant women. Studies have found that there is a strong correlation between the retinal microvessel caliber and hypertension [22–24]. Applying digital retinal photography and imaging software, Wong et al. [22] measured the width of the retinal vessels to objectively quantify extensive arteriole stenosis. In 838 binocular retinal vessel images, they found that the increase in blood pressure was significantly negatively correlated with the diameter of the retinal arterioles. For every 10 mmHg increase in the mean arterial blood pressure, the mean diameter of small arteries in both the eyes decreased by 4.0 *μ*m. Von Hanno et al. [23] and Lee et al. [25] also viewed the mean arterial pressure as the main factor causing changes in the diameter of the retinal arterioles and venules. This reveals that acute hypoxia and ischemia owing to hypertension have a significant impact on microcirculation and retinal thickness and that there is a correlation between microcirculation and retinal thickness. Additionally, the severity of vessel abnormalities is positively correlated with blood pressure. The higher the blood pressure, the more apparent the alterations and the more severe the degree of retinopathy [26, 27]. Kaliaperuma et al. [26] observed that diastolic blood pressure has a significant positive correlation with retinopathy in severe preeclampsia (sPE) patients. A cross-sectional study in Singapore discovered that for every 10 mmHg increase in mean arterial pressure during pregnancy, the caliber of small retinal arteries was significantly reduced [28].

### 2.5. Hypertensive Choroidopathy

The choroid is the corresponding part of the eye that undergoes ischemia in microcirculation disorders in PE. The manifestations of choroidal ischemia generally precede abnormalities of the retinal microvessels. Prior studies identified the presence of HLA class I self-peptides, ICAM-1, and carbonic anhydrase 4 (CA4) in the choriocapillaris, which serves as a regulator of the metabolic and inflammatory environment in the choroid and its supporting tissues [29–31]. As the innermost vascular network, the choriocapillaris provides oxygen and nutrients to the outer retina. Compared with other capillaries, those in the choriocapillaris have a larger lumen diameter. There are circular openings on the capillaries, which are covered by diaphragms, mainly participating in material exchange through subcellular endothelial structures, such as pits, transendothelial channels, window openings, and intercellular connections for material exchange [32, 33]. However, how the choriocapillaris maintains perforated endothelial cells remains unknown, as the subretinal space is generally the only potential space. Once the vascular endothelial cells of the choroid are affected by systemic hemodynamics, the RPE of the outer retina is damaged. The RPE is the transmission channel between the choroid and the photoreceptor cells. A reduced blood supply means hypoxia of photoreceptor cells and visual impairment. Moreover, the RPE has the ability to maintain the homeostasis of the subretinal space [34, 35]. Therefore, the choroid plays an essential role in the proper functioning of the retina.

Fluorescein angiography (FA) and indocyanine green angiography (ICGA) have previously been used to assess choroidal function during pregnancy in PE patients [36–38]. As early as 1980, Mabie and Ober [36] found that FA in PE patients showed delayed choroidal capillary filling. Fastenberg et al. [39], Sathish and Arnold [40], and Hage et al. [41] recorded the changes in angiographs in patients with SRD combined with PE, showing delayed perfusion of the choroidal capillaries, nonperfusion areas, and late slow fluorescein leakage between the subretinal and pigmented epithelial spaces. The above studies suggest that severe impairment of the choroidal vasculature triggers destruction of the blood-retinal barrier, leading to SRD. Despite the fact that angiography is the gold standard for diagnosis, it has not been widely performed in pregnant women due to its invasiveness.

Scholars have carried out related research on pregnant women with PE for quantitative and qualitative analyses of choroid thickness and choriocapillaris flow. The characteristics of the included studies are shown in Table 1. A meta-analysis of 1227 pregnant women demonstrated that the choroidal thickness of the normal pregnancy group was significantly higher than that of the nonpregnant group [42]. It was speculated that this may be related to the increasing circulating volume or alterations in hormone levels during pregnancy. The choroidal thickness of PE pregnant women and healthy pregnant women is compared in several studies by optical coherence tomography (OCT) [43–45]. Researchers found that the choroid thickness of PE women was thinner than that of healthy pregnant women. The area of choroidal capillary flow in the PE group was lower than that of healthy pregnant women, which is likely related to the vascular tissue edema caused by choroidal hyperperfusion or to vasospasm, where the placenta is invaded, but this needs to be confirmed. However, Kim et al. [46] and Benefica et al. [47] found that the choroid of patients with PE was significantly thicker than that of nonpregnant and healthy pregnant women. The choroidal thickness of patients with sPE was the highest. Considering that both studies only screened patients with sPE before labor, if the vessels are severely contracted, microvascular ischemia and hypoxia of the whole body will exacerbate choroidal permeability. Therefore, further research is needed to confirm whether there is a significant difference in choroidal thickness between PE and healthy pregnant women. Using optical coherence tomography angiography (OCTA) to quantitatively monitor choriocapillaris flow has gained popularity in recent studies. Chanwimol et al. [48] found that the choriocapillaris flow was not significantly different between pregnant women and nonpregnant women. However, Urfalıoglu [49] analyzed the choriocapillaris flow area (CBPA) in PE pregnant women, healthy pregnant women, and control nonpregnant women. The outcome indicated that CBPA was higher in both groups of pregnant women than in the control group of nonpregnant women. Currently, the quantitative analysis in the choroidal vascular network remains limited, but it has critical significance for the basic graded diagnosis and treatment of pregnant women.

### 2.6. Hypertensive Retinopathy

The retinal thickness and total macular volume of healthy pregnant women increases significantly in the second and third trimesters [50, 51], which may be ascribed to the increased blood flow and pregnancy-related fluid retention. The included studies of retinal parameters in PE patients are shown in Table 2. Neudorfer et al. [52] stated that the average thickness of the retinal nerve fiber layer (RNFL) around the optic disc was greater in patients with PE. They speculated that the thickening of the RNFL in patients with PE may be because the central nervous system is also involved in pathophysiological changes. The latest study showed that the thickness of the RNFL around the optic disc was higher in the group of women with PE and the group of healthy pregnant women than in the control group [53].

However, other scholars believe that acute severe hypoxic injury of the PE system may change the microcirculation, leading to a permanent reduction in retinal thickness [54, 55]. Kara et al. [56] stated that the RNFL thickness of PE patients was lower than that of normal pregnant women. Arab et al. [57] found that women with sPE or eclampsia had the greatest decrease in the average RNFL thickness around the optic disc. Therefore, it can be inferred that elevated blood pressure causes irreversible damage to the microstructure of the retina, even though there are no obvious clinical macroscopic symptoms. The expression of the tumor necrosis factor (TNF-*α*) and vascular endothelial growth factor (VEGF) is upregulated in the retina of PE patients [58, 59]. As an effective molecule of inflammation, angiogenesis, and apoptosis, TNF-*α* induces a series of pathophysiological changes, such as injury and apoptosis of endothelial and retinal cells, angiogenesis, and vascular leakage, by producing VEGF [60]. Angiotensin II (Ang II) also stimulates endothelial cells to generate VEGF, which has been confirmed to exist in the retina [61]. VEGF is an essential cellular molecule involved in the occurrence of retinal vascular disorders. By motivating the tyrosine kinase receptors VEGFR1 and VEGFR2, VEGF changes the extracellular matrix, destroys the endothelial blood-retinal barrier, promotes the differentiation, proliferation, and migration of endothelial cells, and ultimately, causes retinal impairment.

Saito et al. [62] first used OCTA to observe the retina in PE patients. Newman et al. [53] included 98 pregnant women: 55 in the PE group and 43 in the healthy pregnant group. The data suggested that the superficial foveal density (SFD) and deep foveal density (DFD) of PE patients were lower than those of normal pregnant women, and the DFD was lower than the SFD. There was no significant difference in the foveal avascular zone (FAZ). Urfalioglu et al. [49] found that the area of blood vessels around the optic nerve was significantly reduced compared with that of the control group, reflecting the lack of substantial blood flow around the optic nerve in PE patients. This may be related to the mechanism of hypoperfusion or vasoconstriction generated by hyperperfusion injury. The authors also believe that the self-regulation ability of the optic nerve regarding peripheral circulation is weaker than that of the retinal arterioles and that it responds more strongly to the influence of systemic blood pressure fluctuations. Due to the sample size limitations of the abovementioned studies and patient inclusion criteria, there was no visible retinopathy. Consequently, whether the measurements of the SFD, DFD, and FAZ vary with the severity of the disease remains to be confirmed.

### 2.7. SRD

SRD is a complication of PE, first proposed by von Graefe in 1855. SRD occurs in 1% of PE patients and approximately 10% of eclampsia patients. Most scholars believe that SRD is attributed to choroidal ischemia. When edema and necrosis of the RPE develop, the outer blood-retinal barrier is destroyed, causing choroidal leakage under the retina and forming a serous separation. SRD complicated with PE is mostly reported in the form of case reports [47, 63]. Altalbishi et al. [63] not only reported a case of a pregnant woman with limited SRD but also summarized the fundus changes in PE patients after secondary SRD. The visual prognosis with retinal detachment secondary to PE is usually good, but irreversible tissue damage and necrosis can still occur in the outer retinal and choroidal layers. Therefore, timely diagnosis and intervention cannot be neglected.

### 2.8. CSC

It is well known that pregnancy is one of the risk factors for CSC. Compared with that of patients with normal blood pressure, the vascular system of PE patients includes increased systemic vascular permeability. Choroidal ischemia impairs the RPE function of fluid transport. In addition, subretinal fluid accumulates due to increasing choroidal vascular permeability, accounting for serous nerve sensory detachment. There are few reports describing the prevalence of CSC in women with PE [27, 37, 64]. In a retrospective observational study of 1881 Japanese women, it was believed that the risk of CSC in PE pregnant women was greater in normal pregnancies, especially in women with blood concentrations that were considered to be associated with a higher CSC risk [65].

## 3. Conclusion

The fundus changes in PE patients mentioned above are usually reversible, and the symptoms can be relieved and recovered after termination of the pregnancy or childbirth; but clinically, approximately 1/3 of patients have ocular sequelae [66, 67]. There is conclusive evidence that retinopathy is essential for the prognosis of future cardiovascular events.

Conclusively, fundus alterations in PE are closely related to the disease progression. With advancements in technology and equipment, screening of the eye has become increasingly refined, and the study of structural changes of the fundus, including changes affecting the blood vessels, choroid, and retina, has become the mainstream. However, through the above findings, most of the existing literature is limited to the study of the choroid, and research on the macula, optical disc, and retina needs to be further developed and perfected. Measuring quantitative alterations can be used to prevent severe complications and predict the risk of future hypertension in PE patients, which provide good guidance for the early prevention of the disease. Therefore, the microscopic measurement of fundus data and quantification of the correlation with PE have become the target direction of our next phase of research.

## Figures and Tables

**Table 1:** Quantitative analysis of choroidal thickness.

Study	Location	Eyes (*n*)			Age (year)			Gestational age (week)	Choroidal thickness (*μ*m), mean ± SD		
		Preeclampsia	Normal pregnancy	Nonpregnancy	Preeclampsia	Normal pregnancy	Nonpregnancy		Preeclampsia	Normal pregnancy	Nonpregnancy
Sharudin, 2020	Malaysia	50	50	50	32.5 ± 4.2	31.2 ± 4.1	30.9 ± 5.3	33.9 ± 3.7	557.8 ± 10.7	560.6 ± 15.5	539.3 ± 16.0
Duru, 2016	Turkey	32	41	—	29.59 ± 5.43	27.54 ± 5.25	—	31.81 ± 2.89	351.97 ± 22.44	389.73 ± 49.64	—
Kim, 2015	Korea	7	14	21	31.86 ± 4.06	31.43 ± 2.88	30.43 ± 4.15	34.57 ± 16.81	389.79 ± 25.13	274.23 ± 29.30	264.95 ± 21.03
Sayin, 2015	Turkey	33	40	46	30.5 ± 7.6	28.1 ± 6.2	30.2 ± 6.2	29.7 ± 5.5	333.8 ± 55.3	368.6 ± 67.6	334.8 ± 59.9
Garg, 2014	United States	15	—	19	32.7 ± 7.6	—	27.9 ± 5.1	32.5 ± 4.9	425 ± 90	354 ± 140	363 ± 83
Atas, 2014	Turkey	27	25	26	30.81 ± 6.46	27.76 ± 5.21	29 ± 7.1	38.35 ± 3–.1	353.47 ± 49.37	387.2 ± 60.76	322.35 ± 63.89
Benfica, 2019	Brazil	47	27	—	28.3 ± 6.7	28.1 ± 7.0	—	32.6 ± 3.4	346.7 ± 14.9	318.1 ± 15.6	—
Asal, 2019	Turkey	30	30	—	28.1 ± 6.5	26.6 ± 5.9	—	35.7 ± 2.9	208.5 ± 47.8	200.5 ± 49	—

**Table 2:** Quantitative measurement of retinal parameters.

Study	Location	Eyes (*n*)			Age (year)			Gestational age (week)	Measured areas		
		Preeclampsia	Normal pregnancy	Nonpregnancy	Preeclampsia	Normal pregnancy	Nonpregnancy				
Atas, 2014	Turkey	27	25	26	30.81 ± 6.46	27.76 ± 5.21	29 ± 7.1	38.35 ± 3.1	RNFL (*μ*m) Mean ± SD	Preeclampsia	102.45 ± 8.69
										Normal pregnancy	104.77 ± 10.64
										Nonpregnancy	97.62 ± 12.27

Neudorfer, 2014	Israel	20	—	—	NA	—	—	NA	RNFL (mm)	Mild preeclampsia	91
										Severe preeclampsia	98

Arab, 2018	Iran	210 (70/140)	88	—	28.6 ± 6.2	26.2 ± 5.7	—	36.5 ± 2.7	RNFL (*μ*m) Mean ± SD	Mild preeclampsia	104 ± 10
										Severe preeclampsia	104 ± 23
										Normal pregnancy	106 ± 9

Asal, 2019	Turkey	30	30	—	28.1 ± 6.5	26.6 ± 5.9	—	35.7 ± 2.9	RNFL (*μ*m) Mean ± SD	Preeclampsia	101.7 ± 12.8
										Normal pregnancy	103.3 ± 11.6

Ciloglu, 2019	Turkey	55	43	38	30.05 ± 5.75	31.4 ± 5.15	33.37 ± 7.96	35.89 ± 2.88	RNFL (*μ*m) Mean ± SD	Preeclampsia	103.73 ± 11.61
										Normal pregnancy	104.85 ± 11.95
										Nonpregnancy	97.82 ± 9.78

Garg, 2014	United States	15	—	19	32.7 ± 7.6	—	27.9 ± 5.1	32.5 ± 4.9	Retinal macular volume (mm^3^)	Preeclampsia	9.0 ± 0.4
										Normal pregnancy	8.7 ± 0.5
										Nonpregnancy	8.8 ± 0.4

Kim, 2015	Korea	7	14	21	31.86 ± 4.06	31.43 ± 2.88	30.43 ± 4.15	34.57 ± 16.81	Central subfield retinal thickness, CSRT (*μ*m)	Preeclampsia	262.29 ± 12.39
										Normal pregnancy	258.93 ± 18.97
										Nonpregnancy	264.48 ± 18.73

Kolenko, 2019	Khabarovsk	42	20	—	NA	NA	NA	NA	Macular retinal volume, MRV (mm)	Mild preeclampsia	6.8 ± 0.4
										Average preeclampsia	7.4 ± 0.5
										Severe preeclampsia	8.5 ± 0.7

NA, not available.
